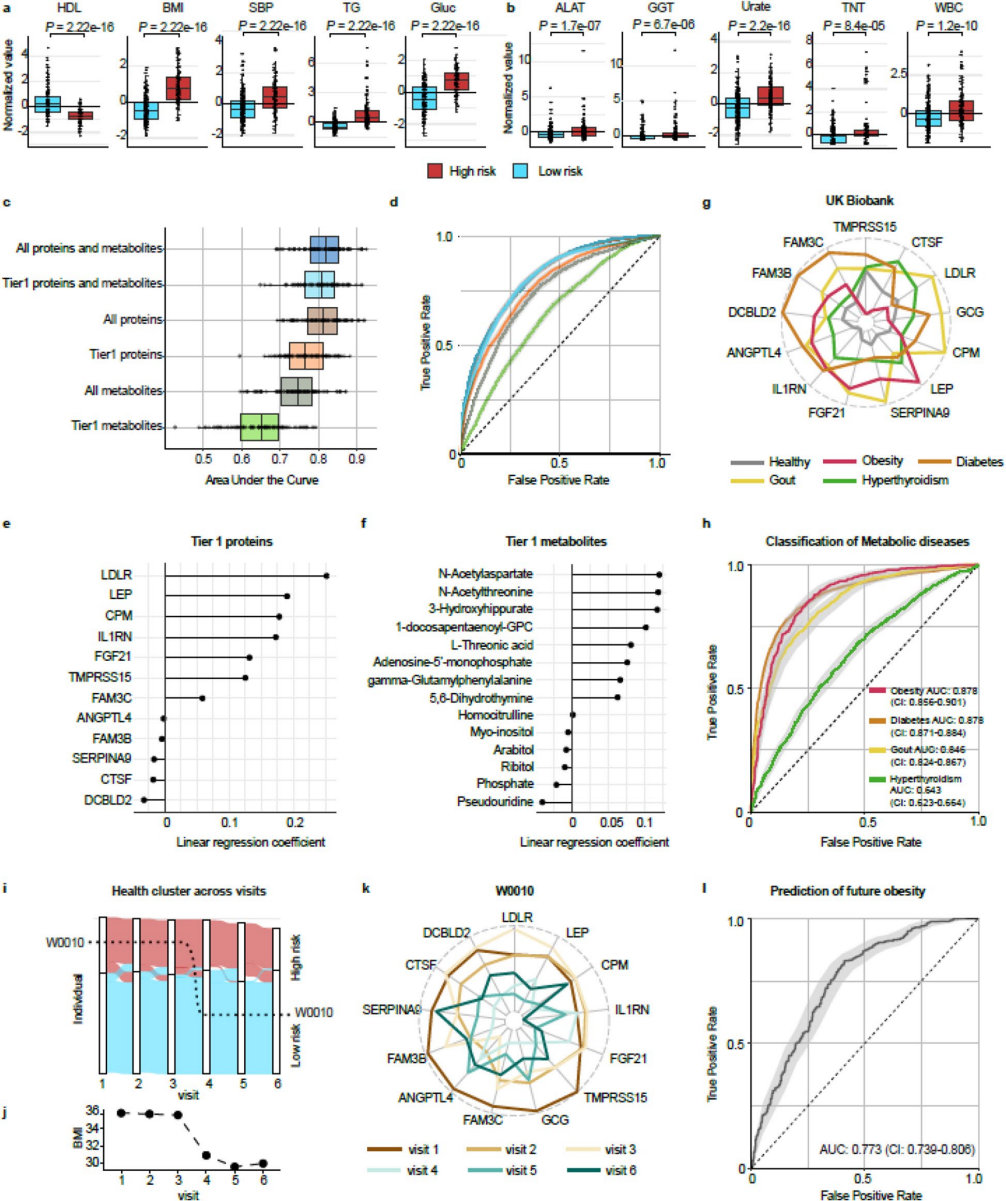# Correction: Longitudinal analysis of genetic and environmental interplay in human metabolic profiles and the implication for metabolic health

**DOI:** 10.1186/s13073-026-01705-y

**Published:** 2026-07-06

**Authors:** Jing Wang, Alberto Zenere, Xingyue Wang, Göran Bergström, Fredrik Edfors, Mathias Uhlén, Wen Zhong

**Affiliations:** 1 Department of Biomedical and Clinical Sciences (BKV), Linköping University, Linköping, SE-581 83 Sweden; 2 Science for Life Laboratory, Linköping University, Linköping, Sweden; 3 Department of Molecular and Clinical Medicine, Institute of Medicine, Sahlgrenska Academy, University of Gothenburg, Gothenburg, Sweden; 4 Department of Clinical Physiology, Sahlgrenska University Hospital, Region Västra Götaland, Gothenburg, Sweden; 5 Department of Protein Science, Science for Life Laboratory, KTH Royal Institute of Technology, Stockholm, Sweden

**Correction: Genome Med 17, 68 (2025)**

**https://doi.org/10.1186/s13073-025-01492-y**

The original publication of this article [1], contained an incorrect version of figure 4 & 7 due to a version error. In figure 4 panels f & g were revised and combined into a single panel f, the results from a HeLa cell line were included alongside the 293T cell line data. In figure 7 panel h was updated with the missing ROC curve for gout. The incorrect and correct figures are shown in this correction article, the original article has been updated.

**Incorrect figure 4** 
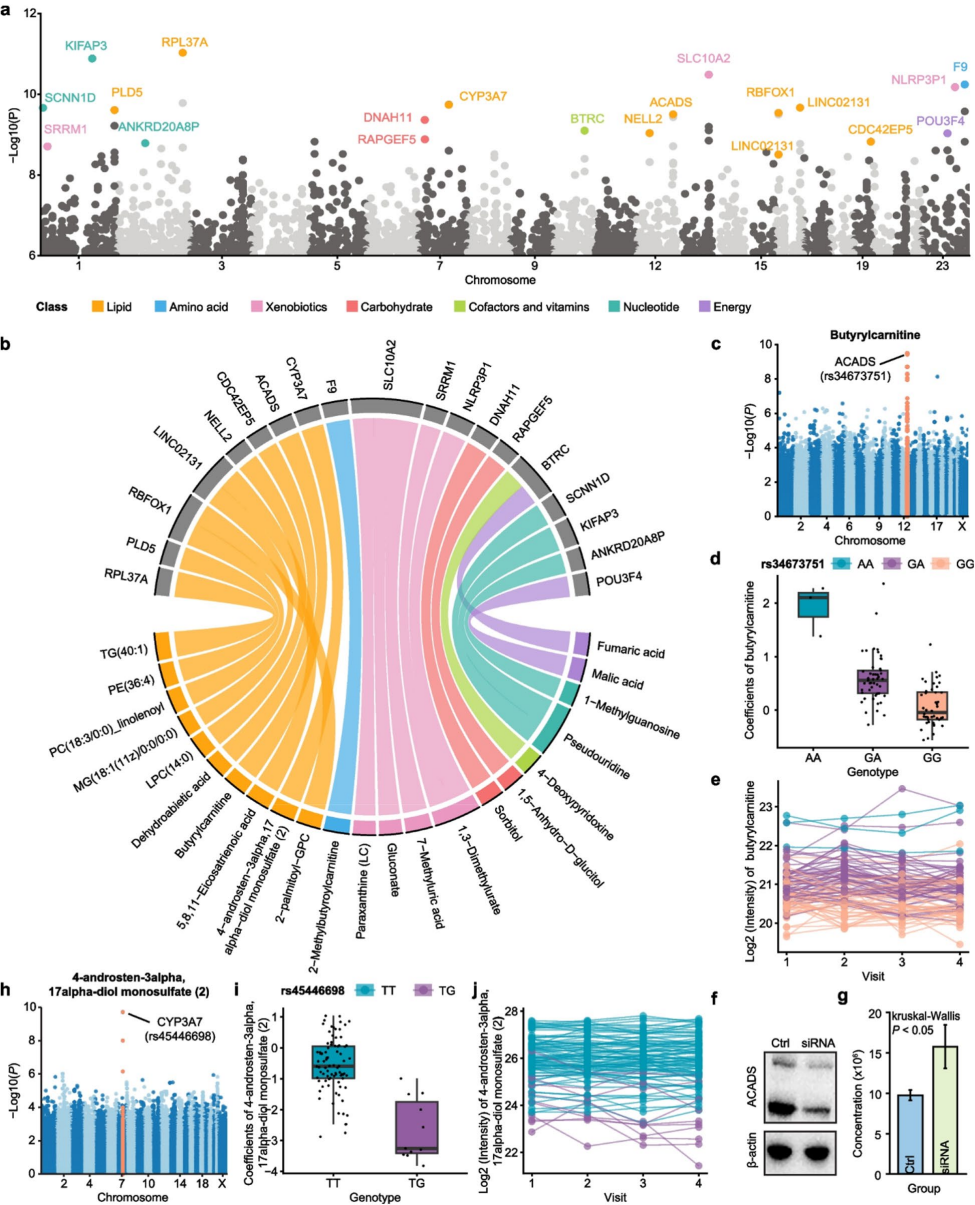


**Correct figure 4** 
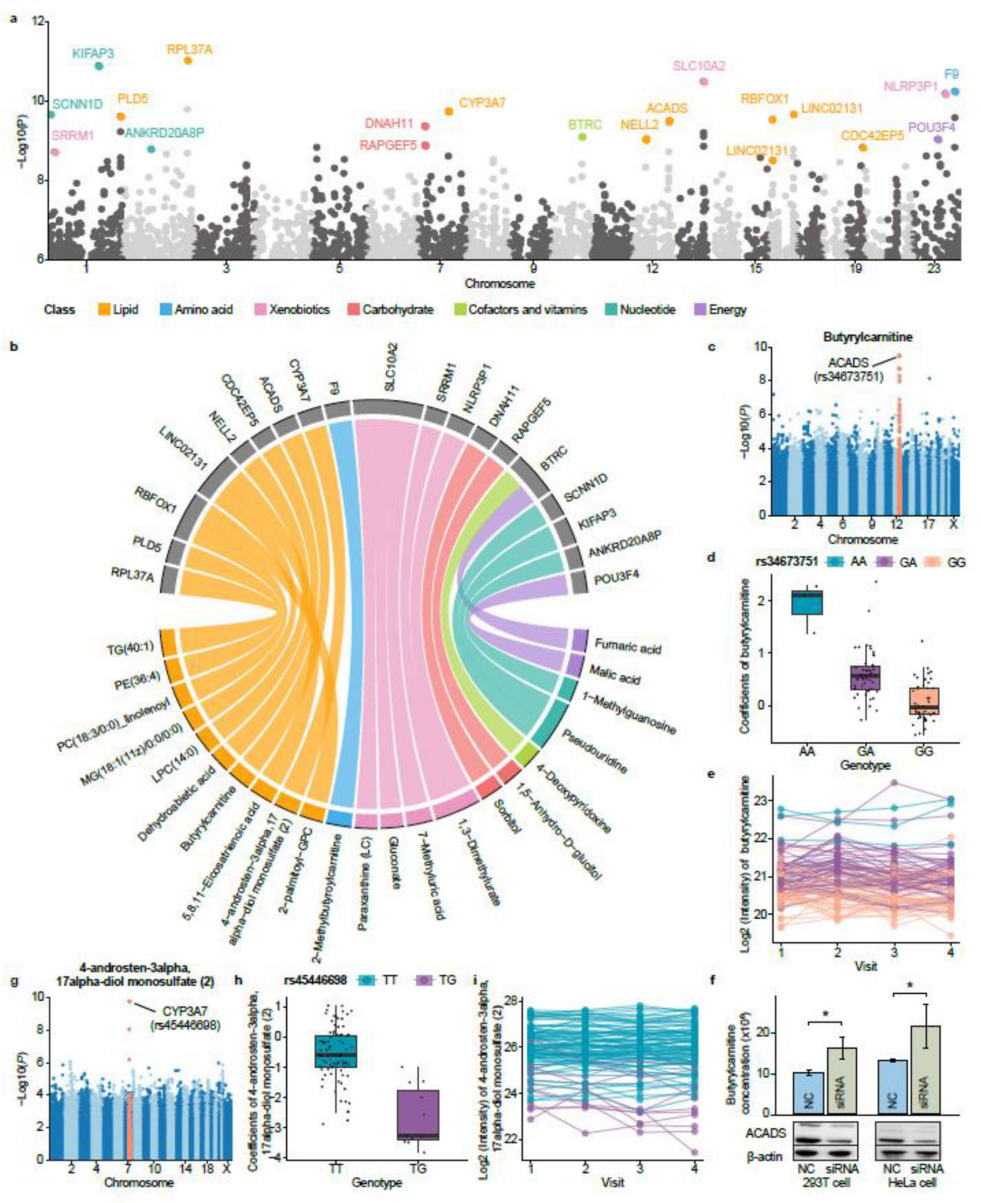


**Incorrect figure 7** 
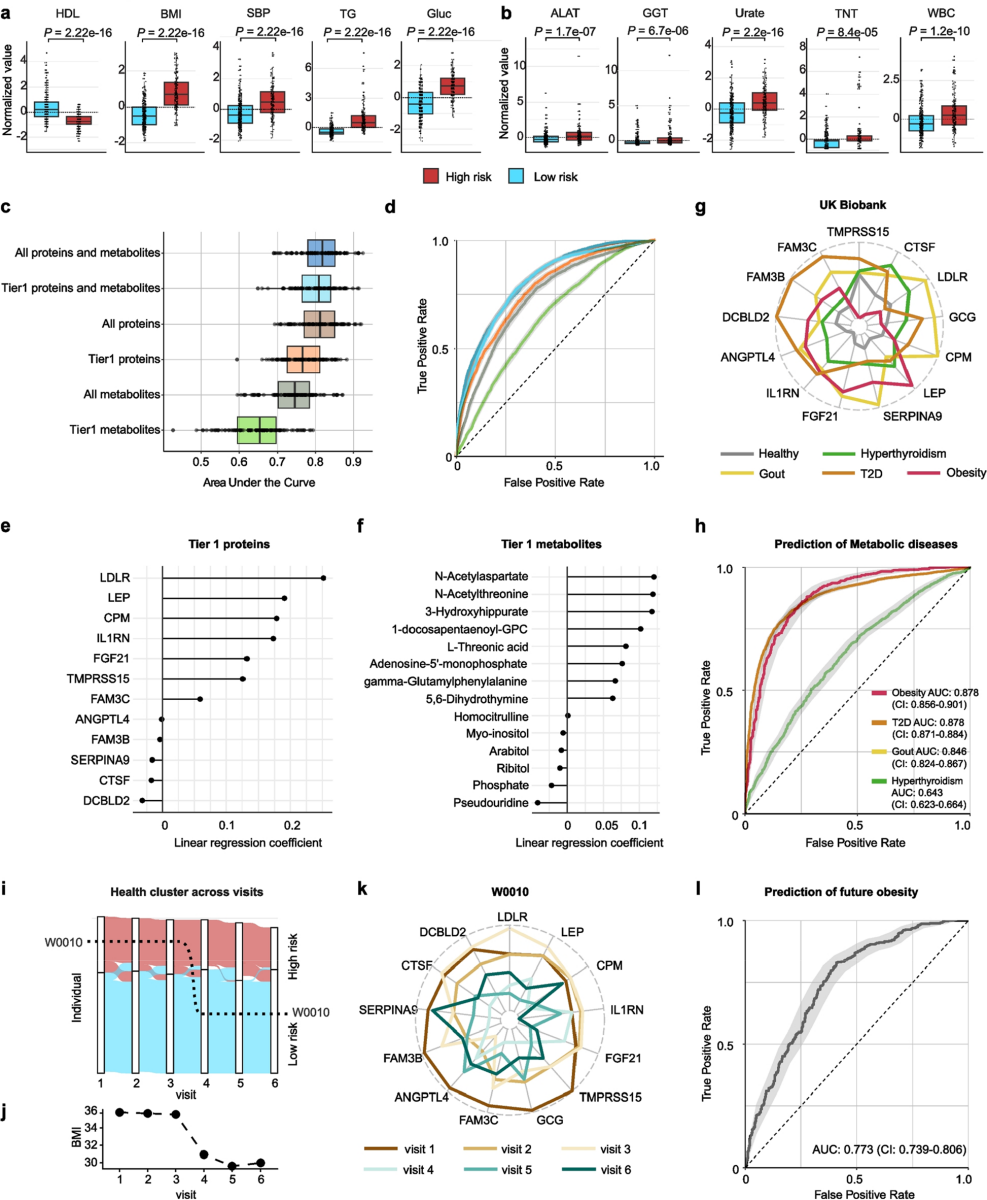


**Correct figure 7**