# Supplementary material for: Longitudinal analysis of genetic and environmental interplay in human metabolic profiles and the implication for metabolic health
Source: Genome Med. 2025 Jun 17;17:68. doi: 10.1186/s13073-025-01492-y (PMC12172340; doi:10.1186/s13073-025-01492-y)
Supplement: Supplementary file 1 — Additional file 1: Figure S1. Flowchart outlining the selection criteria for identifying individuals with metabolic diseases in the UK Biobank cohort. Figure S2. Clustering of 527 analyzed metabolites. Figure S3. Seasonal variation analysis of plasma metabolites and proteins. Figure S4. CCK-8 cell viability assay comparing siRNA and negative control groups. Figure S5. Mendelian randomization protein-metabolite network. Figure S6. Associations between Tier 1 proteins and metabolic diseases. Figure S7. Associations between Tier 1 proteins and obesity. Figure S8. Prediction of future obesity in the UK Biobank. [file 13073_2025_1492_MOESM1_ESM.pdf]

Supplementary Materials for

Longitudinal analysis of genetic and environmental interplay in human metabolic profiles and the implication for metabolic health

Jing Wang[1#], Alberto Zenere[1#], Xingyue Wang[1#], Göran Bergström[2], Fredrik Edfors[3], Mathias Uhlén[3], Wen Zhong[1*]

Supplementary method

Experimental validation

293T cell line was cultured in DMEM (C11885500BT, gibco), supplemented with 10% fetal bovine serum (A5669701, gibco), 1x penicillin-streptomycin (E607011-0100, BBI), in a humidified atmosphere of 5% CO2 at 37ºC. HeLa cell line was cultured in DMEM(C11885500BT, gibco), supplemented with 10% fetal bovine serum (A5669701, gibco), 1x penicillin-streptomycin(E607011-0100, BBI), in a humidified atmosphere of 5% CO2 at 37ºC.

ACADS short interfering siRNA1, GGAGTTGTTTCCCATTGCA and negative control siRNA (Beijing Tsingke Biotech) were purchased from Beijing Tsingke Biotech Co., Ltd. siRNA was diluted to a concentration of 100 μM. Firstly, cells (7×105 per well) were seeded in 6-cm plates and incubated at 37°C for 24 h. A total of 1.6 μL siRNA (50 nM) was added into 200 μL jetPRIME® buffer, and 8 μL jetPRIME® reagent (101000046, Polyplus) was added to the mix. Next, the transfection mix was incubated for 10 min at room temperature. The transfection mix was added to the cells in 3 ml complete medium dropwise and incubated at 37°C for 6-8 h. After 6-8 h, the supernatant was removed and fresh complete medium was added, and the following experiments were performed at 48 h after transfection. The supernatant of 2×106 cells were collected from cell lysis at 48 h after transfection. The protein concentration in the supernatant was determined by Pierce™ BCA Protein Assay Kit. Equal amount of lysis buffer was added to 4-20% FuturePAGETM (ET15420LGel, ACE) and gel electrophoresis was performed. The lysates in the gel were then transferred onto 0.2uM PVDF membranes (ISEQ00010, Merck) for 30 mins. After the transfer, the membrane was blocked with 5% skimmed milk powder and

incubated for 30 min at room temperature. The membrane was washed with TBST, then incubated with relevant primary antibodies (ACADS, 16623-1-AP, proteintech; β-actin, 81115-1-RR, proteintech) for overnight at 4°C. The secondary antibody (Goat anti-Rabbit IgG (H+L) HRP, nitrogen, 31460) was added onto this membrane at a dilution of 1:10000 and incubated at room temperature for 2 h. The membrane was added the ECL chemiluminescent liquid (34580, Thermo Fisher) and then placed into the imaging system for exposure. The relative content of butyrylcarnitine from 2 million cells was detected by a liquid chromatography-quadrupole time-of-flight mass spectrometer (LC-QTOF MS, Agilent #1290-6546).

Supplementary figures

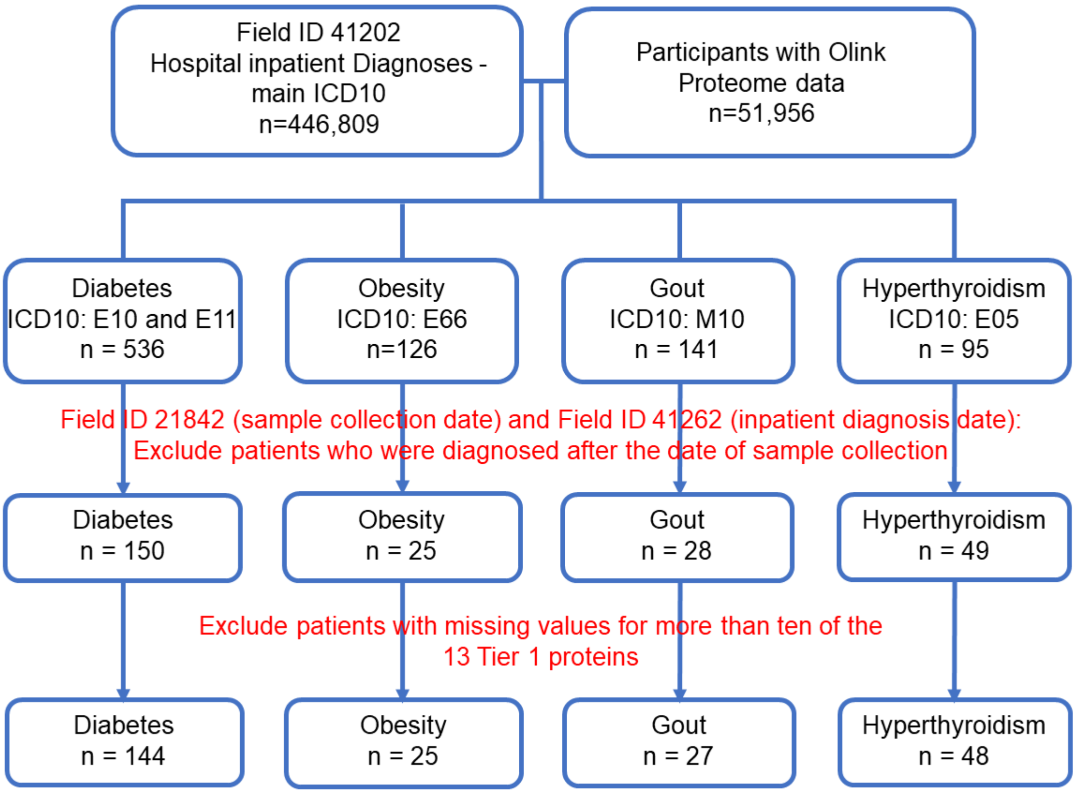

Figure S1. Flowchart outlining the selection criteria for identifying individuals with metabolic diseases in the UK Biobank cohort.

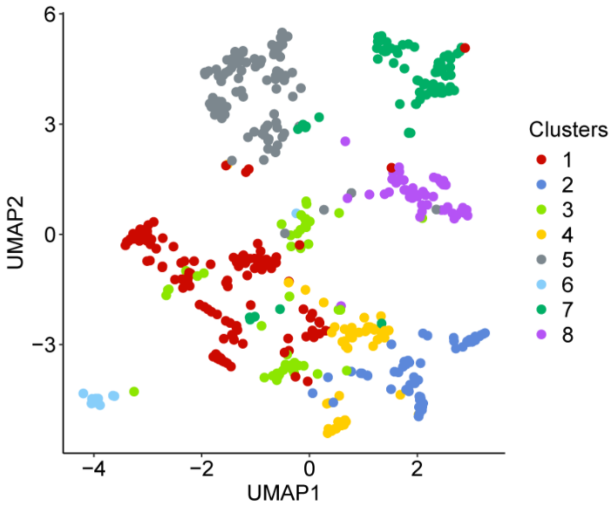

Figure S2. Clustering of 527 analyzed metabolites.

UMAP clustering of the 527 metabolites measured in this study, showing their co-variation patterns, colored by clusters.

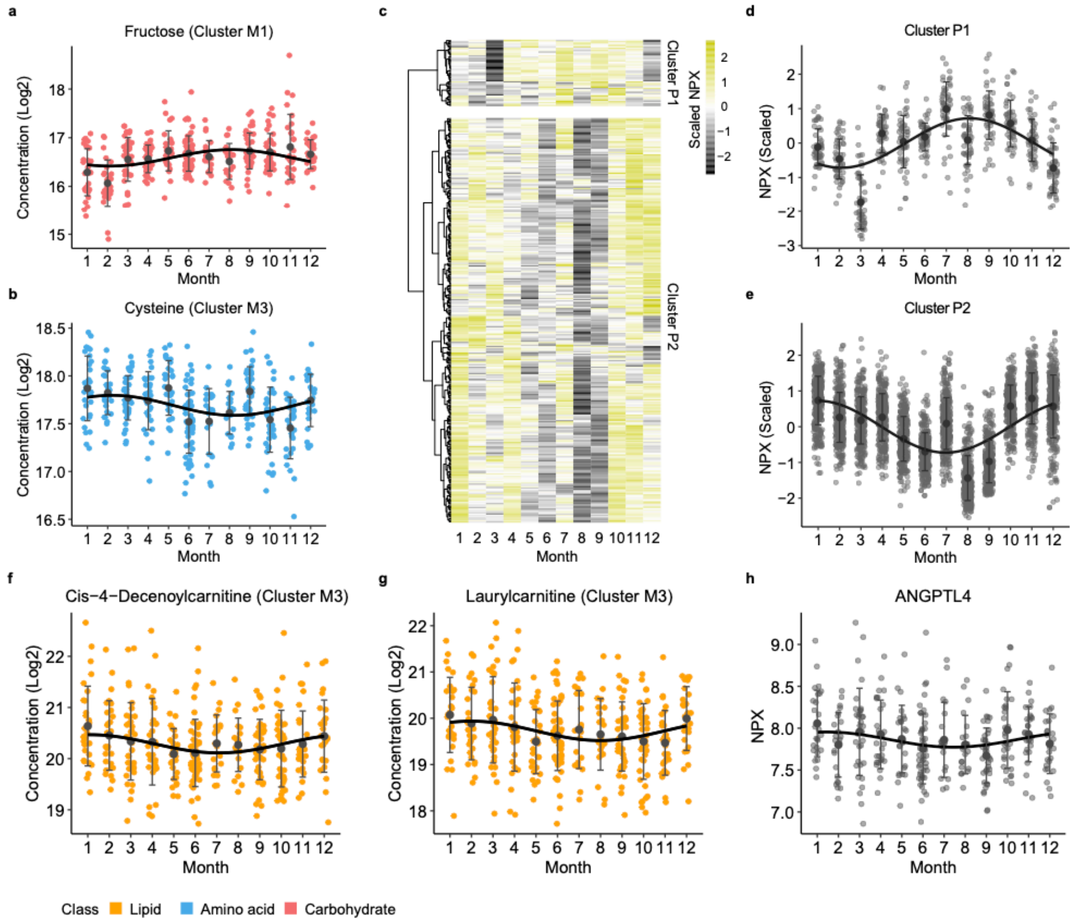

Figure S3. Seasonal variation analysis of plasma metabolites and proteins.

a, b, f, j Scatter plots showing the plasma levels of fructose (**a**) and cysteine (**b**), and cis-4-decenoylcarnitine (**f**) and laurylcarnitine (**g**) across 12 months during the study period, color-coded by the metabolite classes. **c**, Heatmap showing plasma levels of 363 proteins with significant seasonal variations (adjusted $P < 0.05$, Kenward-Roger approximation with Benjamini-Hochberg correction). The plasma levels of proteins in each month were colored based on the scaled NPX values. **d, e** Scatter plots showing plasma metabolite levels throughout the year for proteins in Cluster P1 and P2. **h** Scatter plot showing the plasma levels of ANGPTL4 (angiopoietin-like protein 4) across 12 months. The regression curves were added in **a, b, d-h** by fitting trigonometric functions; and mean and standard deviation (SD) at each month were plotted.

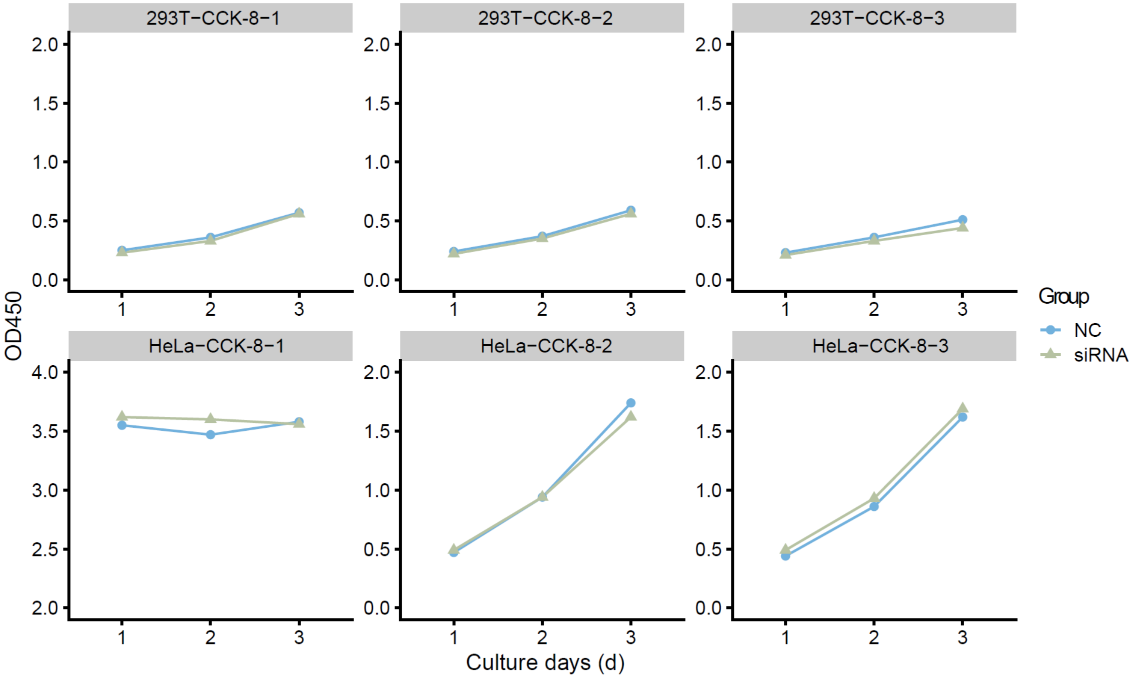

Figure S4. CCK-8 cell viability assay comparing siRNA and negative control groups.

Compare the cell viability of siRNA group and negative control (NC) group in 293T cells and HeLa cells, respectively. Three replicates were performed in each cell line.

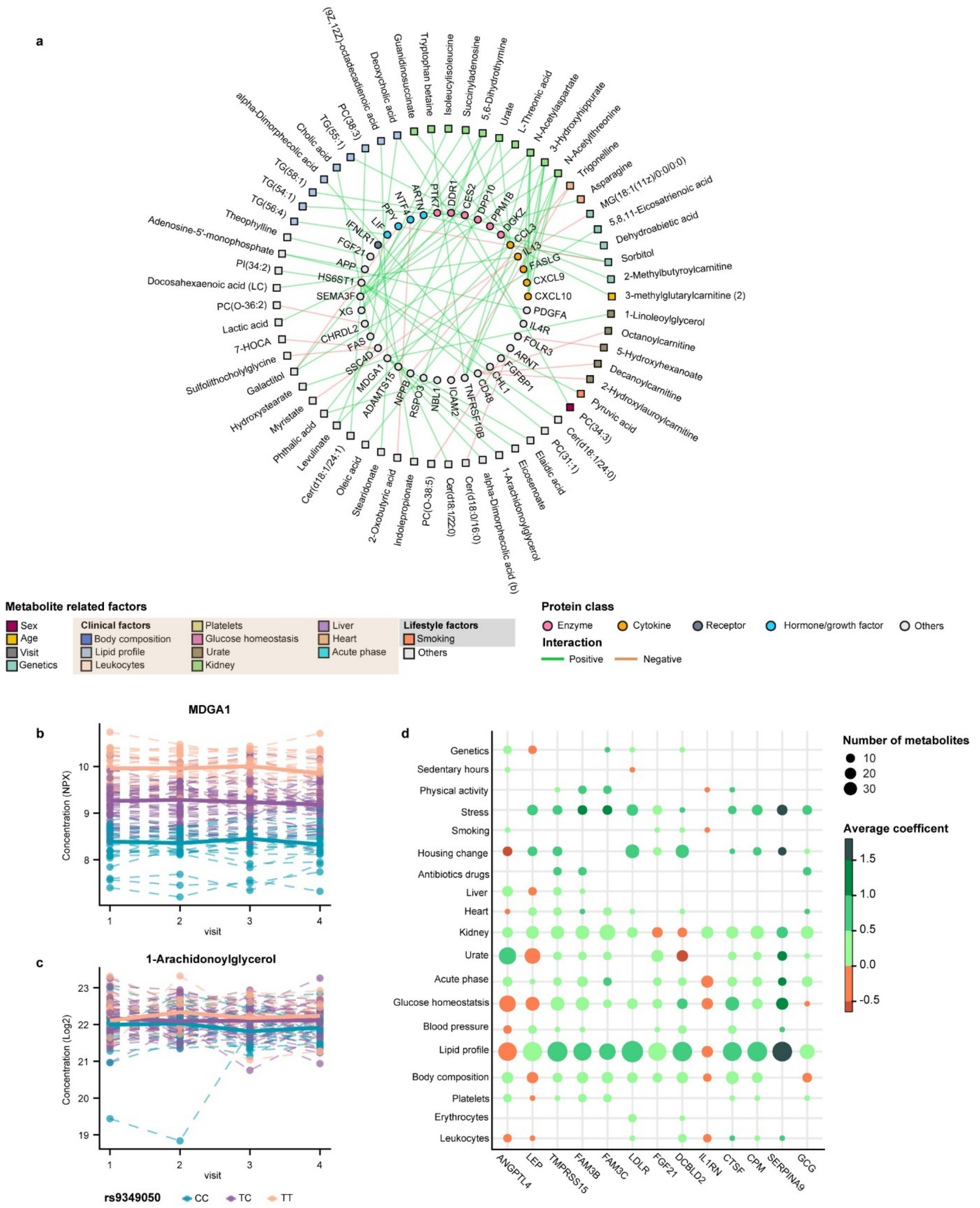

Figure S5. Mendelian randomization protein-metabolite network.

a Network representing all the significant (Benjamini Hochberg adjusted *P*<0.05) protein-metabolite interactions with genetic associations identified by Mendelian randomization (MR) analysis. Solid circles represent proteins in the inner ring, color-coded by protein annotation. Squares represent metabolites in the outer ring, color-coded by different metabolite-related variables. Pairs of related proteins and metabolites are connected by solid lines (indicating correlations supported by both the linear mixed model (LMM) and MR). Green lines indicate positive correlations between proteins and metabolites in the LMM, while red lines indicate negative correlations. **b, c** Longitudinal levels of MDGA1(**b**) and 1-Arachidonoylglycerol (1-AG) (**c**) across study visits, with each individual connected with a dashed line. The median molecular levels were shown by the regression curves, color-coded by genotypes. **d** Bubble plot showing the numbers of metabolites associated with both Tier 1 proteins and genetic, clinical, and lifestyle variables. Color represents the average coefficients from the linear regression.

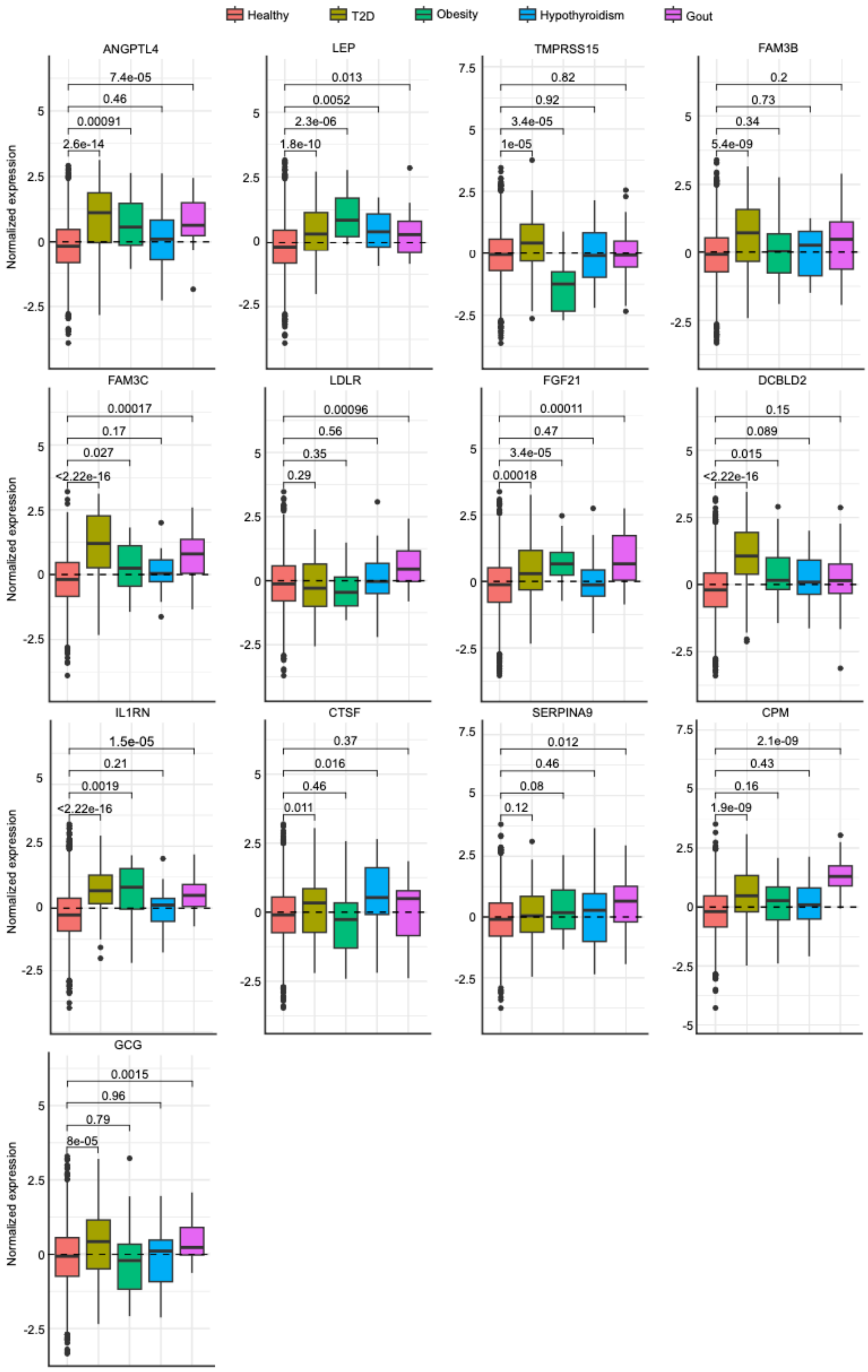

Figure S6. Associations between Tier 1 proteins and metabolic diseases.

Boxplot showing the levels of Tier 1 proteins in individuals with metabolic diseases, including diabetes, obesity, hypothyroidism, and gout, compared with healthy individuals, based on data from the UK Biobank.

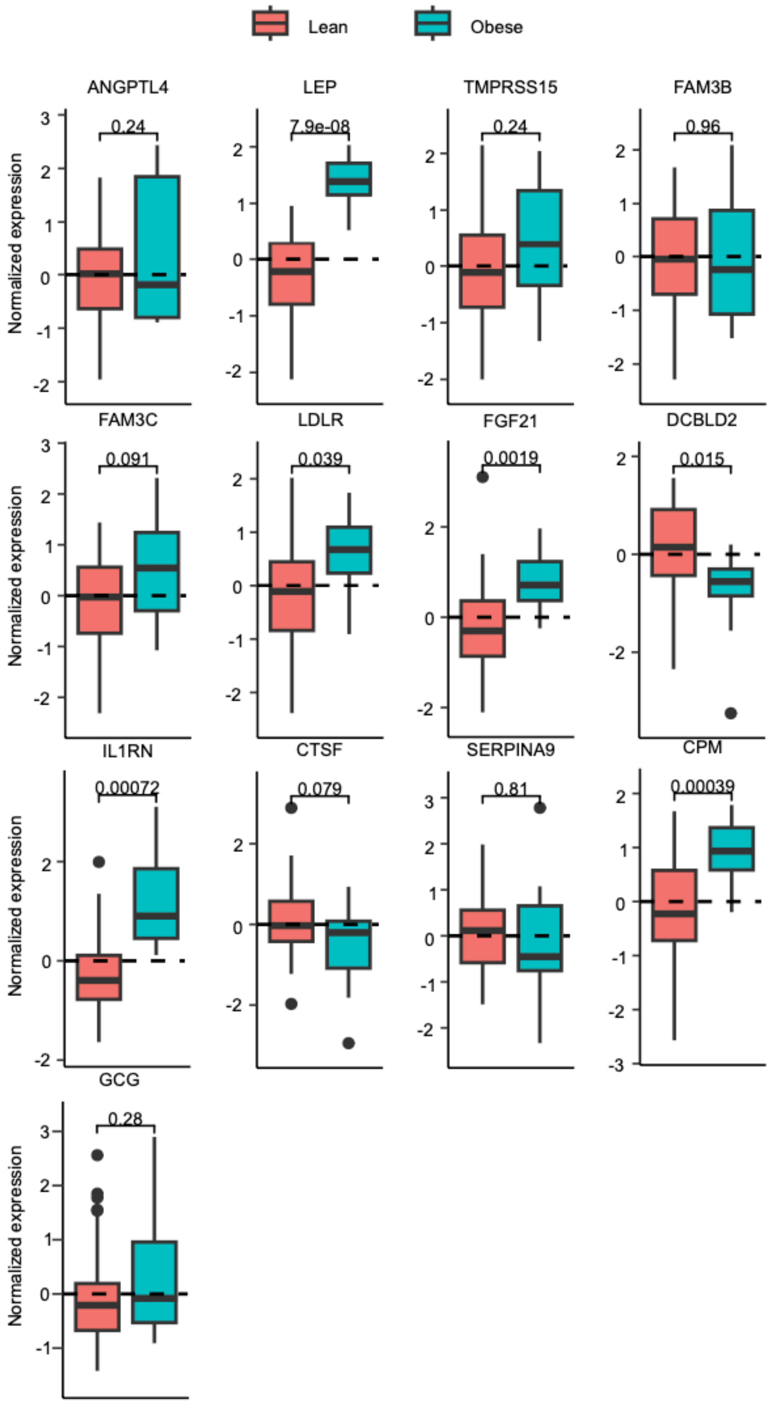

Figure S7. Associations between Tier 1 proteins and obesity.

Boxplot showing the expression levels of Tier 1 proteins in obese individuals (BMI > 30) compared to lean individuals (BMI < 25) based on data from the S3WP study.

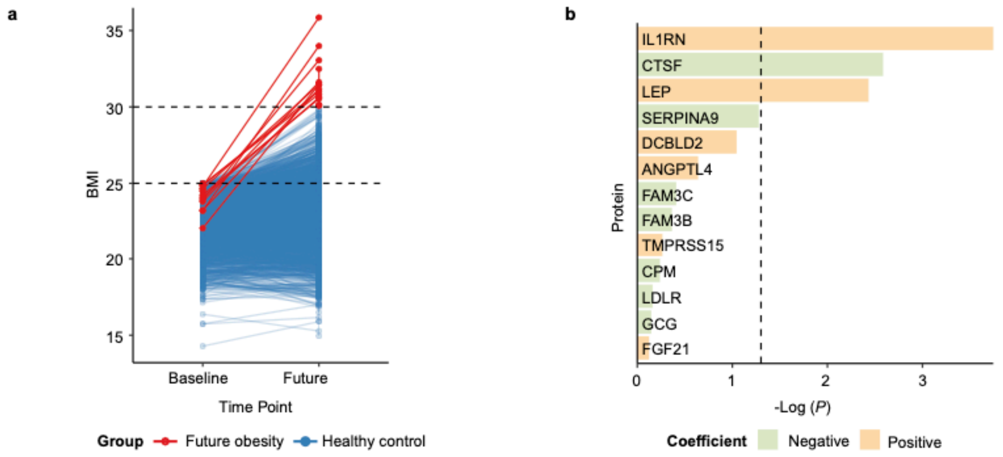

Figure S8. Prediction of future obesity in the UK Biobank.

a Line plot showing the maximum BMI changes of individuals in the UK Biobank from baseline measurements during the study visits. Colors indicate whether an individual had a BMI > 30 at any of the subsequent visits. **b** Bar plot showing the coefficients of Tier 1 proteins for their associations with future obesity using a linear model. *P*-values were calculated by ANOVA with Benjamini-Hochberg correction for multiple testing.
